# *Lippia javanica* (Burm.f.) Spreng.: Traditional and Commercial Uses and Phytochemical and Pharmacological Significance in the African and Indian Subcontinent

**DOI:** 10.1155/2017/6746071

**Published:** 2017-01-01

**Authors:** Alfred Maroyi

**Affiliations:** Department of Botany, University of Fort Hare, Private Bag X1314, Alice 5700, South Africa

## Abstract

*Lippia javanica *occurs naturally in central, eastern, and southern Africa and has also been recorded in the tropical Indian subcontinent. The potential of* L. javanica* as herbal or recreational tea and herbal medicine and its associated phytochemistry and biological properties are reviewed. The extensive literature survey revealed that* L. javanica* is used as herbal tea and has ethnomedicinal applications such as in colds, cough, fever, malaria, wounds, diarrhoea, chest pains, bronchitis, and asthma. Multiple classes of phytochemicals including volatile and nonvolatile secondary metabolites such as alkaloids, amino acids, flavonoids, iridoids, and triterpenes as well as several minerals have been identified from* L. javanica*. Scientific studies on* L. javanica* indicate that it has a wide range of pharmacological activities which include anticancer, antiamoebic, antidiabetic, antimalarial, antimicrobial, antioxidant, antiplasmodial, and pesticidal effects. Although many of the traditional uses of* L. javanica* have been validated by phytochemical and pharmacological studies, there are still some gaps where current knowledge could be improved.* Lippia javanica *is popular as both herbal and recreational tea, but there is need for more precise studies to evaluate the safety and clinical value of its main active crude and pure compounds and to clarify their mechanisms of action.

## 1. Introduction

**Figure 1:**
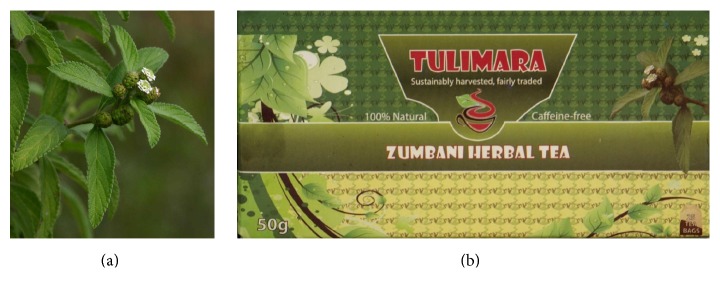
(a)* Lippia javanica *(Burm.f.) Spreng. flowers and leaves (photo: BT Wursten) and (b)* L. javanica* herbal tea traded as Zumbani in Zimbabwe (http://specialityfoods.co.zw/product/zumbani/).

*Lippia javanica *(Burm.f.) Spreng. (Verbenaceae) (Figure 1(a)) has a long history of traditional uses in tropical Africa as indigenous herbal tea or tisane (Figure 1(b)), refreshing beverage, or food additive based on its perceived health and medicinal properties.* Lippia javanica *is rich in volatile oil, particularly caryophyllene, carvone, ipsenone, ipsdienone, limonene, linalool, myrcene, myrcenone, ocimenone,* p*-cymene, piperitenone, sabinene, and tagetenone [1–5]. Research by Viljoen et al. [3] revealed that the essential oil profiles of* L. javanica *are characterized by inter- and intraspecies variations because they are produced by different metabolic pathways. Using cluster analysis, Viljoen et al. [3] identified five chemotypes of* L. javanica *in South Africa and Swaziland, myrcenone-rich type (36–62%), carvone-rich type (61–73%), piperitenone-rich type (32–48%), ipsdienone-rich type (42–61%), and linalool-rich type (>65%). The relative proportion of the chemical constituents of* L. javanica *essential oil is important as this determines the biological properties of the species chemotypes.

*Lippia javanica* belongs to the* Verbena* or vervain family (Verbenaceae) comprising approximately 32 genera and 840 species [97]. The genus* Lippia* L. is named after Augustin Lippi (1678–1701), an Italian botanist and natural historian who was killed in Ethiopia at the age of 23 [98]. Phylogenetic relationships within family Verbenaceae demonstrated that genus* Lippia* and other closely related genera, namely,* Aloysia* Paláu,* Lantana* L., and* Phyla* Lour., are not monophyletic [97]. The boundaries separating these four genera are historically weak, with many taxonomic researchers including species belonging to* Aloysia, Lantana,* and* Phyla* in the genus* Lippia* [99].* Lippia *and* Lantana* genera are the most difficult to separate, as species of these two genera show similarities in their inflorescences that are spicate, often subcapitate during anthesis and elongating in fruit and pedunculated [98]. According to de Campos et al. [100], the genus* Lippia* comprises about 200 species of herbs, shrubs, and small trees distributed throughout south and central America and tropical Africa. Only 15* Lippia *species have been recorded in tropical Africa [101]. The specific name “javanica” was given by the Dutch Botanist Nicolaas Laurens Burman (1734–1793) in 1768, who mistakenly thought that the type specimen was collected in Java, Indonesia [102]. He placed the species in the genus* Verbena, *and the German botanist Sprengel (1766–1833) transferred Burman's name to the genus* Lippia* in 1825 [103].* Lippia javanica* is morphologically similar to* L. scaberrima *Sond. but is much taller and its bracts are shorter than the flowers (Figure 1(a)), while* L. scaberrima* has many stems arising from ground level and is usually less than 0.5 metres high, and its bracts are not longer than the flowers [104].

*Lippia javanica* is an erect woody perennial herb or shrub of up to 4.5 m tall, with strong aromatic leaves which give off a lemon-like fragrance when crushed [105]. Stems are brownish, usually erect or spreading with short stiff tubercle-based whitish hairs and small glands, and branched with inflorescences in nearly all axils. Leaves are opposite or in whorls of 3, blades lanceolate to oblong and densely pubescent, rounded and then cuneate at the base, and crenate-serrate or closely serrulate on the margins except near the leaf base [106]. Flowers occur in conical or oblong spikes, purple or dull-reddish in fruit, dark brownish on drying [101, 106]. The flowers are sessile or with short peduncles, lower bracts of spikes ovate and upper bracts smaller, pubescent, glandular, and densely spreading [101]. The calyx is 2-lobed, half as long as the corolla, pubescent, and densely spreading. The corolla is white, yellowish-white to greenish (Figure 1(a)), usually with a yellow throat, glandular and pubescent outside in the upper half, tube narrowly funnel-shaped from a narrow base [101, 106]. Anthers are sulphur-yellow and nutlets are brown and half ovoid [101, 106].

**Figure 2:**
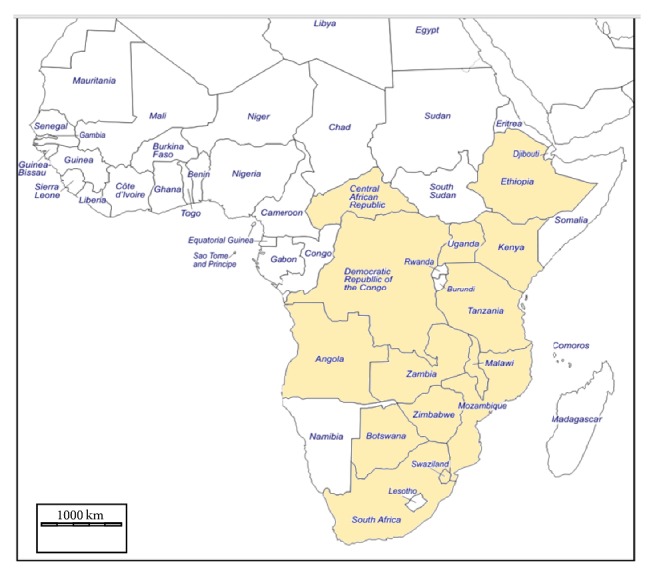
*Lippia javanica* naturally occurs in central, eastern, and southern Africa.

*Lippia javanica *occurs naturally in central, eastern, and southern Africa (Figure 2) and has also been recorded in the tropical Indian subcontinent [12, 13, 23, 39, 47, 61]. In sub-Saharan Africa, the species is native to Angola, Botswana, Central African Republic, Democratic Republic of Congo, Ethiopia, Kenya, Malawi, Mozambique, South Africa, Swaziland, Tanzania, Uganda, Zambia, Zanzibar, and Zimbabwe.* Lippia javanica *has been recorded in low to high altitude (0–2350 m above sea level) woodlands and wooded grasslands, scrub bushland, and grassy rocky kopjes, in riverine vegetation, and on margins of dambos and swampy ground, sometimes on termite mounds, in montane grasslands, and on evergreen forest margins, also in disturbed ground beside roads, forest clearings, plantations, and cultivated land and becoming a weed in derived rangelands [101, 106]. This shows that the plant is highly adaptable to a wide range of climatic, soil, and vegetation conditions.

## 2. Traditional Uses of* Lippia javanica*

**Figure 3:**
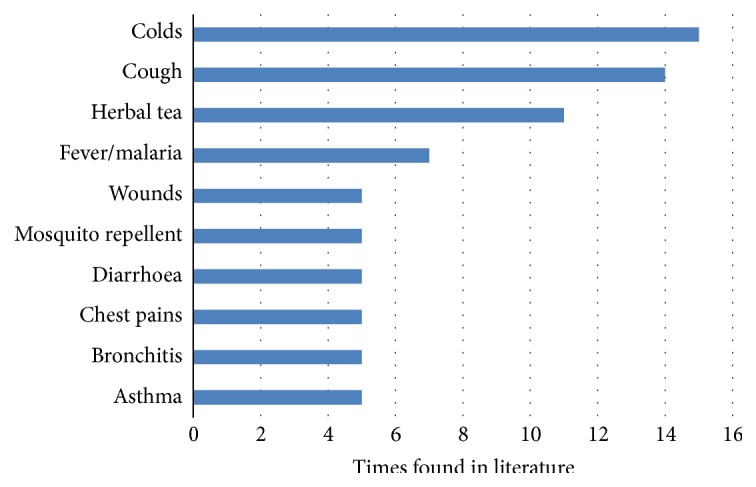
Main ethnobotanical applications of* Lippia javanica* in central, eastern, and southern Africa. An ethnobotanical use is counted only once per publication.

*Lippia javanica *is used for a wide variety of traditional uses (Table 1). Based on literature, the most important traditional applications include its uses as herbal tea and ethnomedicinal applications for (in descending order of importance) colds, cough, fever or malaria, wounds, repelling mosquitos, diarrhoea, chest pains, bronchitis, and asthma (Figure 3). These different uses are discussed in the following seven sections: Food Uses; Respiratory Problems; Gastrointestinal Diseases; Fever, Malaria, and Insect Repellent; Wounds, Injuries, Pain, and Skin Infections; Ethnoveterinary Uses; and Other Uses.

### 2.1. Food Uses

Leaves and twigs of* Lippia javanica* are used as food additives in Kenya [6] and leafy vegetable in India [12, 13].* Lippia javanica* is popular as herbal tea, particularly in Botswana, South Africa, and Zimbabwe [7–11].* Lippia javanica* herbal tea is prepared by steeping fresh or dried leaves, stems, or twigs in boiling water and letting them stand for two to five minutes to release flavour, with or without milk and sugar added according to taste. A stronger brew, known as a decoction, is prepared by boiling fresh or dried leaves, stems, or twigs for more than five minutes. The herbal tea prepared from* L. javanica *leaves, stems, or twigs has a lemon or vanilla aroma and is often used as a common tea (i.e.,* C. sinensis*) substitute or a few leaves, stems, or twigs are added to* C. sinensis* to provide a lemon or vanilla aroma. According to Sõukand and Kalle [87], herbal tea or tisane is an English term used to denote a decoction or infusion made of herbs for medicinal purposes. When* L. javanica* is used as medicinal herbal tea, it is consumed for a limited number of days to treat a specific condition like asthma in Zimbabwe [14], bronchitis in South Africa [19], chest pains in Zimbabwe and South Africa [14, 24], colds in Kenya, South Africa, and Zimbabwe [19, 20, 28–30], cough in South Africa and Zimbabwe [19, 20, 30, 53], and other diseases (see Table 1). From literature, it seems* L. javanica *herbal tea evolved over the years from medicinal tea decoctions or infusions to nonmedicinal uses, where the herbal tea is now drunk for recreation and enjoyment. According to Shikanga et al. [7],* L. javanica *tea is appreciated throughout its distributional range as a general health tonic and also because it is naturally caffeine-free and has a calming and relaxing effect. Research done by other workers, for example, Manenzhe et al. [2], Shikanga et al. [7], Parrant [8], Olivier et al. [81], Motlhanka and Makhabu [9], and Bhebhe et al. [10, 11], revealed that* L. javanica* is a popular recreational tea in southern Africa, consumed on a daily basis within a food context, while medicinal infusions or tisanes of* L. javanica* are taken for a specific medical purpose. During the past 20 years, the use of herbal teas has increased globally [107] because of their functional properties and consumer interest in the health promoting properties of such beverages [108]. The other advantage associated with* L. javanica* usage is that the leaves can be sun-dried and later boiled in water and drunk as herbal tea [9].

Most of* L. javanica* used as herbal tea in rural and periurban communities in central, eastern, and southern Africa is still collected from the wild, although small-scale cultivation has become necessary as it is marketed as herbal tea in Botswana under the brand name “Mosukudu” or “Mosukujane” [109] and in Zimbabwe as “Zumbani” (Figure 1(b)). Although considerable quantities of* L. javanica *are sold in local markets in Botswana and Zimbabwe and also traded on the Internet, there is no data on present production levels, traded volumes, values, and export figures in these two countries. Research by Whiteside [110] showed that sales of* L. javanica *tea bags generated an income of R20300.00 (US$5718) during 1994/1995 in Botswana. There is increased demand for* L. javanica* herbal tea especially in the light of growing health consciousness worldwide, with the estimated potential demand for the species and its products around 100 tonnes per year on the local market and 1000 tonnes per year on the export market (http://bio-innovation.org/work/fever-tea-tree/). Considering the rapid growth of the herbal tea industry worldwide and the increasing demand for* L. javanica *products, there is need for the improvement of* L. javanica *products as well as development of new products. Therefore,* L. javanica* has potential to make the transition from limited local use to commercial and international product.* Lippia javanica* has been identified as one of the few plant species that should be integrated in the domestication process in farming systems in sub-Saharan Africa to support medicinal, nutritional, and income security of local communities [107, 111]. According to Van Wyk [111],* L. javanica* is of commercial value as herbal tea and health drinks. Therefore, commercialization of* L. javanica *is unlikely to be viable if the product is sorely harvested from the wild. In the same line commercialization of* L. javanica *might be more worthy if other products other than tea are derived from the plant.

### 2.2. Respiratory Problems

The different parts of* L. javanica*, for example, the leaves and twigs, are used for the treatment of asthma, coughs, colds, influenza, pneumonia, tuberculosis, and bronchial problems in Bangladesh, Botswana, Ethiopia, Kenya, South Africa, and Zimbabwe [14–16, 19, 20, 23, 25, 26, 28–32, 53, 112]. In Bangladesh, leaf infusion of* L. javanica* is taken orally mixed with 3-4 pieces of cloves of* A. sativum*, 2-3 times daily as remedy for chest pains [23]. In Ethiopia, leaves of* L. javanica* are chewed with butter as remedy for chest pains and cough [26]. In India,* L. javanica* leaf decoction is taken orally as remedy for respiratory disorders [39]. In Kenya, leaves are sniffed [31] or half glass of hot leaf infusion is taken orally three times a day [28, 29] as remedy for colds and cough. Research done by Davids et al. [19] in South Africa revealed that about 50 g of leaves is added to a cup of boiling water to produce an infusion which is taken orally as remedy for coughs, colds, and bronchial problems or the infusion is applied to the skin or mixed with Vaseline to make an ointment. In South Africa, leaf or twig decoction is taken orally for asthma, colds, and cough [16, 53], leaves are used in washes and poultices for chest pains [25], and leaves are boiled for 5 minutes and one cup of extract is taken orally thrice a day for chest pains and tuberculosis [24, 42]. According to York [18], two handfuls of* L. javanica* leaves are boiled in two litres of water and patient is steamed once or twice a day to treat coughs, chest pain, headaches, fever, chills, a sore throat, or a blocked nose. This decoction can also be taken orally by drinking half a cup daily [18]. Alternatively, a handful of* L. javanica* roots or leaves are mixed with a handful leaves of* A. glabratum* or* B. transvaalensis *or* B. uniflora* or* B. cathartica* or* C. anisata* or* C. brachiata* or* C. molle *or* E. grandis *or* H. kraussii *or* Hypoxis *spp. or* K. mosambicina *or* P. neochilus *or* P. guajava *or* S. serratuloides *or* T. sericea *or* T. riparia *or* T. emetica *to treat blocked nose, chest pain, cough, earache, fatigue, fever, influenza, headache, runny nose, sleepless nights, sore throat, tiredness, and tonsillitis [18]. Leaf and stem infusion of* L. javanica* are taken orally together with* A. afra* by the Zulu people in South Africa as remedy for bronchial ailments, colds, and cough [20]. “*Imbiza,”* a popular herbal decoction prepared from* L. javanica *and* C. obliquus, *is used by the Zulu people in South Africa as herbal tonic and an immune booster and also for the treatment of cancer, chest pains, colds, diabetes, HIV or AIDS symptoms, skin infections, and tuberculosis [27]. In some communities in South Africa,* L. javanica* leaf or root infusion is taken orally as remedy for influenza and malaria [33] and respiratory disorders [40, 41]. In Zimbabwe, leaf or root decoction is taken orally or smoke of burnt leaves and roots is inhaled as remedy for chest pains [14] and leaf and twigs are boiled in water and infusion is taken orally as remedy for colds and cough [30]. According to Gelfand et al. [14], Shona people in Zimbabwe rub leaf ointment of* L. javanica* on the chest and abdomen as remedy for pneumonia and leaf decoction is taken orally and body washed with leaf decoction as remedy for shortness of breath or dyspnoea. In South Africa, a leaf and stem infusion of* L. javanica *are taken orally together with leaves of* A. afra* as remedy for measles [20].

Based on literature, inhalation of smoke from* L. javanica* appears to be a popular remedy for asthma, chest pains, colds, and chronic coughs in Botswana, South Africa, and Zimbabwe [14, 15, 20, 32]. In Botswana,* L. javanica* leaf infusion vapour is inhaled as remedy for colds and cough [32]. In South Africa, for example, leaves and twigs are burned and smoke is inhaled as remedy for asthma and cough [15] or steam from leaf infusions is inhaled or hot leaf infusions are taken orally against colds and cough [20]. In Zimbabwe, smoke of burnt leaves and roots is inhaled as remedy for chest pains [14].

### 2.3. Gastrointestinal Diseases

The leaf and root decoction or infusions of* L. javanica* are used as remedies of the digestive system diseases such as cholera, diarrhoea, and dysentery. For example, in Mozambique, root decoction is taken orally as remedy for a type of diarrhoea commonly known as “chinhamucaca” which is characterized by milky diarrhoea accompanied by vomiting in children [44]. Leaf decoction is used as herbal medicine for diarrhoea in Kenya [113]. In South Africa, the leaves of* L. javanica* are crushed and mixed with cold or hot water and the mixture is then sieved and a quarter of a cup (75 mL) is taken twice a day until diarrhoea subsides [34]. The Venda people in South Africa use leaf infusions as prophylactic against diarrhoea [40]. Research done by Palgrave et al. [37] in South Africa revealed that tea infusions of the* L. javanica* leaves are used as remedies for HIV/AIDS opportunistic infections such as lung infections and diarrhoea. Previous research in Mozambique [114], South Africa [115], and Zimbabwe [116] showed that gastrointestinal disorders, particularly cholera, diarrhoea, and dysentery are among human diseases often treated with herbal medicines. For those patients diagnosed as having intestinal worms in Venda, South Africa, a leaf infusion of* L. javanica *leaves is used as an anthelmintic [40]. Therefore, these findings illustrate that herbal medicines including* L. javanica* can play an important role in basic health care of local communities through treatment and management of cholera, diarrhoea, and dysentery.

### 2.4. Fever, Malaria, and Insect Repellent

Traditionally,* L. javanica *is commonly used to treat fever and malaria and repel insects throughout its distributional range [4, 14, 16, 17, 33–35, 40, 48–50, 58–60]. In South Africa, a decoction of fresh or air dried leaves is used to wash or steam body parts infested with lice [50]. In India, the whole plant is used to repel lice in poultry [61]. In South Africa and Zimbabwe, whole plant or leaves are burnt to repel mosquitoes [4, 34, 35, 48, 49, 58]. In South Africa and Zimbabwe,* L. javanica *is widely used to get rid of ticks and other ectoparasites; for example, ticks are sprayed with crushed leaves mixed with water or twigs are used as bedding in fowl runs [58–60]. Leaf and twig decoction of* L. javanica *are taken orally as remedy for fever [16]. The Venda people in South Africa use leaf infusions of* L. javanica *as prophylactic against malaria [40]. In Zimbabwe, leaf decoction is taken orally as remedy for fever [14].

*Lippia javanica *is also cultivated on a commercial scale by a rural community in Giyani, the Limpopo province, South Africa, for the production of mosquito-repellent candles [117]. Clinical studies using human volunteers showed that* L. javanica* repels no less than 95% of mosquitos, whereas most mosquito repellents repel only 42% of them [118]. Based on these findings, the Council for Scientific and Industrial Research (CSIR), South Africa, signed a benefit sharing agreement with traditional healers allowing for the commercial cultivation of* L. javanica* aimed at establishing an indigenous oil industry for rural development and large-scale production of antimosquito candles and other insect repellents. The mosquito repellent is registered as a patent under the Fertilisers, Farm Feeds and Stock Remedies Act (Act 36 of 1947) as a pest repellent [119]. These findings and the establishment of a large-scale production of antimosquito candles and other insect repellents strengthen the view that* L. javanica* is a potential source of antipesticidal agents and to some extent validate the traditional use of the plant species for insect pest control. Pesticidal plants such as* L. javanica* are increasingly being used as alternatives where synthetic products are unaffordable or are not available or are ineffective. A number of studies have indicated that the use of* L. javanica* as a pesticide is a long-standing tradition passed down from generation to generation [120].

### 2.5. Wounds, Injuries, Pain, and Skin Infections

*Lippia javanica *is used as remedy for a variety of skin infections and injuries in Kenya, South Africa, Swaziland, and Zimbabwe. In South Africa, leaf infusions are used to treat skin disorders, such as boils, chicken pox, febrile rashes, heat rashes, measles, scabies, scratches, and stings [20, 21, 40, 50, 54]. In Kenya, about 50 g of fresh leaves is wrapped around a fresh wound to enhance healing [57] and, in South Africa, leaf infusions are taken orally as remedy for wounds [54]. In South Africa, powder from burnt roots of* L. javanica *is applied to scarifications made around sprained joints to facilitate healing [20]. In Kenya and Zimbabwe, leaf infusion is taken orally to treat measles or a patient is washed with leaf infusion [14, 38]. The Zulu people in South Africa take a mixture of chopped handful leaves of* L. javanica *with the same amount of bark of* A. burkei*,* O. engleri*,* S. birrea*,* S. cordatum*, and* T. elegans* boiled in two litres of water as an enema for the treatment of sores [56]. In Swaziland, about 30 g of* L. javanica *leaves and similar amount of* Acanthospermum australe* Kuntze are boiled in 5 litres of water and decoction is taken as remedy for sores [55]. In Zimbabwe, root ashes of* L. javanica *mixed with fat are applied to the skin of a patient with scabies [14]. Wound healing is a process which involves distinct overlapping phases of coagulation, inflammation, proliferation, and tissue remodelling [121]. The same authors argued that a set of complex biochemical events takes place in a closely orchestrated cascade to repair the wound and any errors in the wound healing process can lead to delayed healing or formation of hypertrophic scars. Abubakar [122] argued that colonization of wounds by opportunistic microorganisms usually delays the wound healing process and/or may lead to infectious condition. Therefore, application of* L. javanica* on wounds and skin infections leads to disinfection, debridement, and provision of a suitable environment for aiding the wound healing process.* Lippia javanica* has potential for therapeutic use in wound and skin diseases management, but there is need for research on the safety, phytochemistry, and biological properties of the species.

### 2.6. Ethnoveterinary Uses

The leaves, stems, twigs, and whole plants of* L. javanica* are used as ethnoveterinary medicine in India, Kenya, South Africa, and Zimbabwe. The Xhosa people in the Eastern Cape province, South Africa, use* L. javanica *leaves for the disinfection of meat that has been infected with anthrax [20]. In Kenya, the stem of* L. javanica* is used to preserve milk by applying it to the gourd before milk fermentation [62]. In India, whole plants are used as lice repellants while, in South Africa and Zimbabwe, crushed leaves mixed with water are used to get rid of ticks and twigs are used as bedding in fowl runs to get rid of ectoparasites [58–60].

### 2.7. Other Uses

*Lippia javanica* is used as a good luck charm, to treat persons experiencing bad dreams, to ward off evil spirits, to protect one from lightening, and to protect the home (Table 1). In Zimbabwe,* L. javanica *leaves are prepared as an infusion to treat persons experiencing nightmares [64]. In both South Africa and Zimbabwe, evil spirits are cleansed by washing the body of an affected person by leaf infusion of* L. *javanica [14, 123]. In Swaziland, 50 g leaves of* L. javanica* and* C. molle* are ground into a powder and 5 litres of water is added and face and hands are washed to remove bad luck when exposed to a corpse [71]. In South Africa, the whole plant is placed on a patient's bed after the circumcision ceremony [58], to prevent odours and freshen surrounding air. A mixture of* L. javanica* leaves and roots is used to clean tools and hands before and after funerals, the stems and leaves are used as brooms to sweep grave sites, and the entire plant is also used when coming from the mortuary to remove bad spirits [58, 124]. The corpse is washed with* L. javanica* infusion after death to prevent odours forming or if the corpse has an odour, women place pieces of* L. javanica* in the nostrils of the corpse and sweep the room with twigs where the person was sleeping [58]. In KwaZulu Natal, South Africa, if the meat started to smell it will be boiled with* L. *javanica leaves to take away the smell [58]. The Xhosa people in the Eastern Cape province, South Africa, use* L. javanica *leaves for the disinfection of meat that has been infected with anthrax [20]. In KwaZulu Natal, South Africa, leaves are sprinkled in toilets to prevent odours [58]. Leaves of* L. *javanica are sprinkled in houses for pleasant smell in the Limpopo province, South Africa [35], and Kenya [31]. In KwaZulu Natal, South Africa, it is believed that a person can repel snakes by placing a small stem with leaves on his or her head [58]. In Malawi, Swaziland, and Zimbabwe, people showing sign of mental disorder, madness, or hysterical outbursts are required to wash their bodies with leaf infusions [14, 67, 68, 71].

## 3. Phytochemical Constituents and Nutritional Composition of* Lippia javanica*

Multiple classes of phytochemicals including volatile and nonvolatile secondary metabolites, such as alkaloids, amino acids, flavonoids, iridoids, and triterpenes as well as several minerals, have been identified from* L. javanica* [1–5, 7, 13, 27, 73, 78, 81, 83–86, 88–94, 120, 123, 125–127]. Leaves, flowers, and twigs of* L. javanica* have a wide variety of the so-called classic nutrients, such as minerals, carbohydrates, proteins, fats, and vitamins (Table 2).* Lippia javanica* leaves are a good source of minerals such as cadmium, calcium, chromium, cobalt, copper, iron, magnesium, manganese, selenium, and zinc [27]. These authors assessed the levels of the elements in* L. javanica* leaves and found the elements to be in the decreasing order of Ca > Mg > Fe > Zn > Mn > Cu > Se > Cr > Pb > Co > Cd for total concentrations and Ca > Mg > Fe > Zn > Cu > Cr > Pb for water extractable forms. These results corroborate an observation made by Sedaghathoor et al. [128] that Ca and Mg are among the most abundant elements in tea plants. These mineral elements are important in human nutrition since* L. javanica* is used as herbal tea and food additive (Table 1). Calcium, magnesium, iron, manganese, and zinc play a major role in activating some enzymes and regulating many responses of cells to stimuli [129]. Some of the mineral elements identified from* L. javanica* leaves are required by the human body for repair of worn out cell tissues and strong bones and teeth and building of red blood cells and other related tissues. Therefore, since* L. javanica* has appreciable concentrations of mineral elements such as calcium, magnesium, iron, manganese, and zinc (Table 2) which are essential for enzyme metabolism, these mineral elements could enhance the nutritional and curative properties of the species.

Bhebhe et al. [10] determined the total phenolic and tannin content and radical scavenging activities of* L. javanica*, comparing it with* Aspalathus linearis* (Burm.f.) R. Dahlgren (Rooibos™), a commercial South African herbal tea, and other popular herbal teas in Zimbabwe (Table 3).* Adansonia digitata* L.,* Fadogia ancylantha* Schweinf.,* Ficus sycomorus* L., and* Myrothamnus flabellifolius* Welw. are indigenous herbal teas consumed in Zimbabwe. The tannin content of* L. javanica* is very low when compared to* A. linearis* (Rooibos) (Table 3).* Lippia javanica *has higher radical scavenging activity than* A. linearis* (Table 3) which is probably due to higher total phenolic content in comparison to* A. linearis* which is a popular herbal tea consumed by 10% of the global herbal tea market [10]. In another study, Bhebhe et al. [11] determined and compared the effect of several solvents, namely, hot water, 50% methanol, ethanol, 50% ethanol, acetone, 50% acetone, and ethyl acetate, on phenolic composition and free radical scavenging activity in common black tea,* C. sinensis,* and five other well-known herbal teas including* L. javanica*. In all the seven solvents used,* L. javanica* had higher total phenolic content than* C. sinensis* implying that* L. javanica* is competitive to the black tea in terms of phenolic content. Shikanga et al. [7] found leaf extracts of* L. javanica* to have higher phenolic content of 14.8 mg/g gallic acid equivalent of dry weight than flowers (9.9 mg/g) and twigs (8.3 mg/g). Phenolic compounds found in plants are known to play an important role as antioxidants in exhibiting the medicinal properties such as antibiotic, anti-inflammatory, anticancer, and antiallergic properties [10, 66, 130, 131].

The compounds isolated from* L. javanica* are documented and listed in Appendix A and their structures are presented in Appendix B. Simple phenolic compounds and caffeic acid and its derivatives are some of the compounds that have been identified in* L. javanica *and examples include coumarin** 1**, 3,4-dihydroxy-*β*-phenylethoxy-O-[4′′-*β*-caffeoyl-*α*-rhamnopyranosyl-(1*‴*,3′′)-O-*β*-glucopyranoside], commonly referred to as verbascoside** 2,** and 3,4-dihydroxy-*β*-phenylethoxy-O-[6′′-*β*-caffeoyl-*α*-rhamnopyranosyl-(1*‴*,3′′)-O-*β*-glucopyranoside] commonly referred to as isoverbascoside** 3** isolated by Olivier et al. [81] from the aerial parts of the species. Nonvolatile diterpenes, known as iridoid-glycosides, have also been isolated from* L. javanica* by Rimpler and Sauerbier [82] represented by theveside-Na** 4** and theveridoside** 5**. Mujovo et al. [83] isolated a long chain alkane “4-ethylnonacosane”** 6** and four flavanones apigenin** 7**, cirsimaritin** 8**, 6-methoxyluteolin 4′-methyl ether** 9,** and 6-methoxyluteolin 3′,4′,7-trimethyl ether** 10** from ethanolic extracts of* L. javanica* leaves. Madzimure et al. [120] identified an array of phenolic glycosides and flavonoids which include crassifolioside** 11**, luteolin** 12**, diosmetin** 13**, chrysoeriol** 14**, tricin** 15**, isothymusin** 16**, eupatorin** 17**, 5-dimethyl noboletin** 18**, genkwanin** 19**, salvigenin** 20,** and an alkaloid xanthine** 22**. Ludere et al. [84] isolated lippialactone** 21** from the ethyl acetate extract of aerial parts of* L. javanica*. Neidlein and Staehle [85] and Dlamini [86] isolated 19 amino acids, compounds** 23** to** 41** in Appendix A, from* L. javanica*. At least 131 different classes of essential oil compounds (compounds** 42**–**172** in Appendices A and B) have been isolated from* L. javanica *by several researchers [3–5, 83, 85, 86, 88–94, 123]. Hutchings and van Staden [96] isolated a toxic triterpenoid saponin, icterogenin** 173,** from* L. javanica* leaves. These different classes of essential oil compounds have been associated with various therapeutic activities such as anaesthetic, analgesic, anti-inflammatory, antimicrobial, cardiovascular, decongestant, digestive, expectorant, hepatoprotective, and sedative activities as well as stimulant of nervous system and tonifying effects [132]. Meanwhile flavonoids possess several pharmacological properties including antibacterial, anticancer, anti-inflammatory, antioxidant, antiviral, and hepatoprotective effects [133] which play important roles in human health. Flavonoid such as apigenin** 7** is reported to possess antibacterial [134] and hepatoprotective [135] properties. Apigenin** 7** and luteolin** 12** are reported to possess anti-inflammatory and analgesic effects [133], affecting the function of enzyme systems involved in the generation of inflammatory processes, especially tyrosine and serine-threonine protein kinases [136, 137]. It has also been reported that apigenin** 7** prevents HIV-1 activation via a novel mechanism that involves inhibition of viral transcription [138] and luteolin** 12** demonstrated synergistic effects with another flavonoid kaempferol against herpes simplex virus [133]. Kamiya [139] documented the importance of essential amino acids such as lysine, valine, isoleucine, and histidine in terms of the risks to health if they are deficient. The author also documented their biological effects which include muscle protein maintenance, potentiation of immune function, tissue repair acceleration after burn or trauma, protecting liver from toxic agents, lowering blood pressure, modulating cholesterol metabolism, and stimulating insulin or growth hormone secretion. Therefore,* L. javanica* leaves and other plant parts which have shown to be rich in flavonoid and polyphenolic compounds, amino acids, and essential oil could play an important role in the treatment and management of diseases such as hypertension and inflammation listed in Table 1.

## 4. Pharmacological Activities

Scientific studies on* L. javanica* indicate that it has a wide range of pharmacological activities (Table 4), which include anticancer [140], antidiabetic [141], antimalarial [4, 49, 142], antimicrobial [2, 3, 7, 17, 73–75, 77, 83, 84, 126, 143], antioxidant [7, 10, 11, 75, 77, 125], antiplasmodial [2, 79, 80, 84, 144, 145], and pesticidal effects [1, 2, 59, 95, 120, 146–150] and cytotoxicity [2, 73, 79, 120] activities. Table 4 summarizes some of the pharmacological studies undertaken on* L. javanica* extracts aimed at evaluating some of the ethnomedicinal uses of the species documented throughout its distributional range (see Table 1). Some of the listed pharmacological activities may not relate directly to the documented ethnomedicinal uses of the species but may provide some insight into the species' potential therapeutic value and bioactive properties and application.

### 4.1. Anticancer Activity

Fouche et al. [140] reported anticancer activity of dichloromethane root extract of* L. javanica* against three human cells, exhibiting TGI value of 1.82 *μ*g/mL for breast MDA-MB-435, 1.86 *μ*g/mL for breast MDA-N, and 2.09 *μ*g/mL for melanoma MALME-3M. Based on literature, a couple of terpenoid compounds that have been isolated from* L. javanica* are known to have antitumor properties. For example, linalool** 120** is known to have antitumor activity which plays a protective role against hepatotoxicity and the compound has anti-inflammatory activities as well [151]. Research by Yang et al. [152] showed limonene** 92** to have inhibitory effect on pancreatic and mammary tumors. Another terpenoid compound, *α*-pinene** 44,** is known to inhibit translocation of NF-*κ*B or p65 protein into nuclei of LPS-stimulated THP-1 cells [153]. These findings serve as a scientific validation for the use of* L. javanica* as a component of a herbal concoction known in KwaZulu Natal province as “imbiza,” prepared by mixing* L. javanica* with* C. obliquus* as herbal medicine for cancer [27].

### 4.2. Antidiabetic Activity

Arika et al. [141] determined the in vivo antidiabetic activity of aqueous leaf extracts of* L. javanica* in white male alloxan-induced albino mice. The aqueous leaf extracts of* L. javanica *at all dose levels significantly lowered the blood glucose levels in both oral and intraperitoneal routes. The antidiabetic effect of* L. javanica *could have been due to the observed presence of flavonoids. The polyhydroxylated flavonol enhances lipogenesis and glucose uptake in the adipocytes and flavanoids have demonstrated insulinmimetic properties as the compound is known to be effective at controlling blood sugar levels. These findings strengthen the view that* L. javanica* is a potential source of antidiabetic agents and to some extent validate the traditional use of the plant species mixed with* C. obliquus* to form a herbal concoction known in KwaZulu Natal province, South Africa, as “imbiza” used against diabetes [27].

### 4.3. Antimalarial Activity

Govere et al. [142] found that topical application of* L. javanica* alcohol extract leads to 76.7% protection against* Anopheles arabiensis* for 4 hours.* Lippia javanica *has been used as a mosquito repellent by the rural communities in Zimbabwe for a long time and previous studies have shown that essential oils from the species have very strong and lasting repellent activity against female* A. arabiensis *[64]. Research by Lukwa et al. [4] revealed that topical application of 5 mg/cm^2^ of* L. javanica *leads to 100% protection against* Anopheles aegypti *for 8 hours. Mavundza et al. [154] screened dichloromethane and ethanol leaf extracts of* L. javanica* for adulticidal activity against* A. arabiensis*. The authors observed dichloromethane and ethanol activities of 45% and 55% mosquito mortality, respectively. These findings strengthen the view that* L. javanica* is a potential source of antimalarial agents and to some extent validate the traditional use of the plant species as mosquito repellent in India [47], South Africa [34, 49, 58], and Zimbabwe [4, 48].

### 4.4. Antioxidant Activity

Leaf infusions of* L. javanica *exhibited antioxidant activity with EC_50_ value of 358 *μ*g/mL and contained 14.8 mg/mL of dry weight gallic acid equivalent phenolic compounds [7]. The EC_50_ value of 358 *μ*g/mL obtained for* L. javanica* by Shikanga et al. [7] compares well to those of many commercial teas, including antioxidant capacity of Rooibos Fresh Pack™ herbal teas* (A. linearis)* with the best antioxidant activity of 333 *μ*g/mL. The high antioxidant activities displayed by* L. javanica* infusions can be partially attributed to the high levels of verbascoside** 2** (1.5 mg/g dry weight) reported by Olivier et al. [81] in the leaf extract of the species. Earlier research by Muchuweti et al. [125] reported 74.4% inhibition of the DPPH radical by an ethanolic leaf extract of* L. javanica*. Lekganyane et al. [75] reported antioxidant activity in* L. javanica *and the acetone extracts of the species displayed antioxidant activity on BEA chromatogram [77]. Bhebhe et al. [10] reported the antioxidant activities of* L. javanica* based on the DPPH, reducing power and inhibition of phospholipid peroxidation assays. Free radical scavenging activity of* L. javanica* is attributed to phenolic compounds since these compounds have an ideal structural chemistry for free radical scavenging activity [155]. Bhebhe et al. [11] determined the effect of several solvents on the free radical scavenging activity of* L. javanica* using the DPPH assay. Free radical scavenging activity expressed as IC_50_ ranged from 0.022 ± 0.001 g/mL to 0.066 ± 0.001 g/mL; see Table 4.

### 4.5. Antiplasmodial Activity

Prozesky et al. [144] evaluated* L. javanica* leaf acetone extract for in vitro antiplasmodial activity using PfUP1, a chloroquine resistant strain of the malaria parasite* Plasmodium falciparum* by means of the flow cytometric test. The IC_50_ value for* L. javanica* was 4.26 *μ*g/mL. Manenzhe et al. [2] evaluated essential oil, piperitenone** 162,** isolated from* L. javanica* for antiplasmodial activity using chloroquine diphosphate as positive control and found it active against a chloroquine sensitive strain of* P. falciparum* in micromolar concentrations with IC_50_ of 8 *μ*g/mL. Clarkson et al. [80] evaluated* L. javanica* roots and stems extracts for in vitro activity against* P. falciparum *using the parasite lactase dehydrogenase (pLDH) assay and chloroquine diphosphate (Sigma) as the positive control. The dichloromethane, methane, and water extracts showed IC_50_ values ranging from 3.8 to >100 *μ*g/mL; see Table 4. Omolo et al. [145] screened the essential oil of* L. javanica* for fumigant toxicity to* Anopheles gambiae* which exhibited LD_50_ of 4.3 × 10^−3^ mg cm^−3^. Ayuko et al. [79] showed that* L. javanica* root extracts have antiplasmodial activity against* P. falciparum* with IC_50_ ranging from 1.35 to 18.59 *μ*g/mL; see Table 4. Lippialactone** 21**, derived from the ethyl acetate extract of aerial parts of* L. javanica,* exhibited some activity against the chloroquine sensitive D10 strain of* P. falciparum* with an IC_50_ value of 9.1 *μ*g/mL and is also mildly cytotoxic [84]. Compared to chloroquine, the compound is approximately 2000 times less active against the D10 strain of* P. falciparum *[84].

### 4.6. Antimicrobial Activities

*Lippia javanica* is widely used in the treatment of a wide range of infectious diseases caused by microorganisms. Viljoen et al. [3] determined the antimicrobial properties of* L. javanica* by evaluating the time kill studies of the species' essential oil using the disc diffusion assay on three respiratory pathogens* Klebsiella pneumoniae*,* Cryptococcus neoformans, *and* Bacillus cereus*. This study showed that the killing rate was greatest for* K. pneumoniae followed by C. neoformans *and very little reduction of microbial populations was observed for* B. cereus*. The efficacy of* L. javanica *oil for* K. pneumoniae *showed a killing rate within 30 minutes for the concentrations 0.25, 0.5, 0.75, and 1%,* C. neoformans *showed a killing rate for concentrations 0.5, 0.75, and 1% within 1 hour, and the lowest concentration of 0.25% took 8 hours before a bactericidal effect was noted while* B. cereus *showed some reduction in colonies [3]. The positive antimicrobial activity of* L. javanica* as revealed by the time kill study could be attributed to linalool** 120** which averages between 65 and 70% in yield [3, 5, 88, 90] and has known antimicrobial properties [93, 156–159]. These findings somehow corroborate the traditional use of* L. javanica *as herbal medicine for a wide range of bacterial and fungal respiratory ailments indicated in Table 1.

Manenzhe et al. [2] evaluated essential oil, piperitenone** 162,** isolated from* L. javanica* for antibacterial activity on cultures of* Bacillus subtilis, Staphylococcus aureus, *and* Escherichia coli *using imipenem, cefazolin, and ampicillin as positive controls. The authors found piperitenone** 162 **to inhibit* S. aureus* and* E. coli *at 1% dilution. Acetone, hexane, and methanol leaf extracts and essential oil isolated from* L. javanica* showed some activity against fifteen Gram-positive and Gram-negative bacteria with MIC values ranging from 1.5 to >12 mg/mL [60]; see Table 4. In a similar study, Samie et al. [73] demonstrated that a pure compound piperitenone** 162 **isolated from* L. javanica* has antibacterial activities against* Acinetobacter calcoaceticus, Micrococcus kristinae, Salmonella typhi,* and* S. aureus* using dimethyl sulphoxide and kanamycin as controls, with MIC values ranging from 12 to 50 *μ*g/mL; see Table 4. Shikanga et al. [7] evaluated the antibacterial activity of* L. javanica *methanolic leaf extract against* S. aureus, Enterococcus faecalis, E. coli, *and* Pseudomonas aeruginosa *using the serial microdilution method with gentamycin (Virbac®) and acetone as positive and negative controls, respectively.* Lippia javanica* displayed antibacterial activities with MIC values ranging from 0.13 to 0.42 mg/mL against all four pathogens; see Table 4. The obtained minimum inhibitory concentrations are promising, since natural products with MIC values below 1 mg/mL are generally considered to be noteworthy findings [160]. Lippialactone** 21**, derived from the ethyl acetate extract of aerial parts of* L. javanica,* exhibited some activity against the* E. coli *and* S. aureus *at a concentration of 10 mg/mL [84]. Lekganyane et al. [75] reported antibacterial activity of* L. javanica* acetone leaf extracts against* E. coli*,* E. faecalis*,* P. aeruginosa, *and* S. aureus *with MIC values ranging from 0.32 to 0.64 mg/mL and total activity of the same species ranging from 127 to 253 mg/mL; see Table 4. Methanol and water leaf extracts of* L. javanica *exhibited some antiproteus activity against* Proteus mirabilis* and* Proteus vulgaris* with MIC values <2000 *μ*g/mL with standard discs of ampicillin (2 *μ*g) and chloramphenicol (10 *μ*g) as positive controls [74]. Some of the antibacterial properties of* L. javanica *can be attributed to the phytochemical constituents of the species; for example, the phenolic compound apigenin** 7** is a well-known antibacterial agent [7, 134]. Apigenin** 7 **was shown to be highly active against* Vibrio cholera* and* E. faecalis* [161], while Basile et al. [162] reported the inhibition of* S. typhi, P. mirabilis,* and* P. aeruginosa *by the compound. Therefore, results from these antibacterial evaluations of* L. javanica* give credence to the use of the species' infusions against bacterial infections and other related diseases.

Shikanga et al. [126] investigated the antifungal activities of leaf extracts and essential oil compounds isolated from* L. javanica* leafy extracts against a Guazatine®-resistant strain of* Penicillium digitatum*. The methanolic leafy extracts, isoverbascoside** 3** and verbascoside** 2 **compounds, isolated from* L. javanica* inhibited fungal growth at concentrations above 0.6 mg·mL^−1^, causing significant inhibition of mycelial growth. Verbascoside** 2** is well-known for its antimicrobial properties and has been found to inhibit viruses, bacteria, and fungi [163]. Similarly, Samie et al. [143] investigated antifungal effects of acetone and hexane leaf extracts of* L. javanica* against* Candida albicans, Candida krusei, *and* C. neoformans* isolated from AIDS patients using the microdilution method with nystatin, Roche, and DMSO as positive and negative controls, respectively. Noteworthy moderate antifungal activities were recorded from* C. krusei* with MIC value of 1.88 mg/mL and other recorded MFC and MIC values for other species ranged from 1.88 mg/mL to >7.5 mg/mL; see Table 4. Thembo et al. [76] investigated the antifungal activity of aqueous and organic extracts of* L. javanica *using a serial microdilution assay. Generally, extracts of* L. javanica* exhibited weak activity; see Table 4.

### 4.7. Antimycobacterial Activity

Mujovo et al. [83] evaluated* L. javanica *compounds against a drug-sensitive strain of* Mycobacterium tuberculosis* using the radiometric respiratory techniques. Of all the isolated compounds, only one triterpenoid carboxylic acid, euscaphic acid** 172,** exhibited antimycobacterial activity with MIC value of 50 *μ*g mL^−1^ against this strain. In a similar study, the leaf extract of* L. javanica* exhibited antimycobacterial activity against* M. smegmatis* in an evaluation which used microdilution assay and rifampicin as control [77]. Acetone extract was the best extractant with MIC value of 0.47 mg/mL; it extracted antibacterial agents which was indicated by the lowest MIC value [77]. Masoko and Nxumalo [77] also evaluated the total activity of* L. javanica *which averaged 13 mL/g suggesting that the extract prepared from one gram of* L. javanica *could be diluted to a volume of 13 mL and will still inhibit* M. smegmatis* efficiently. According to Semenya and Maroyi [115], tuberculosis caused by* M. tuberculosis *is a serious disease requiring effective strategies and tools to control and manage it. Therefore, preliminary evaluations done by Mujovo et al. [83] and Masoko and Nxumalo [77] serve as a scientific validation for the use of* L. javanica* in traditional medicine for treatment of tuberculosis and other respiratory ailments in South Africa [24, 27, 115] and Uganda [43] as well as their efficiency in tuberculosis drug discovery.

### 4.8. Antiviral Activity

Mujovo et al. [83] found that (E)-2(3)-tagetenone epoxide** 42 **and piperitenone** 162** inhibited the HIV-1 reverse transcriptase enzyme by 91% and 53%, respectively, at 100 *μ*g mL^−1^ based on a nonradioactive HIV RT colorimetric ELISA kit. Little is known about the HIV RT activity of* L. javanica *extracts or compounds, but flavonoids are known to be active against viral RT and also as potent inhibitors of the cellular alpha and beta DNA polymerase [83], while luteolin** 12** is active against HIV RT [164, 165].* Lippia javanica* is traditionally used to treat HIV/AIDS symptoms in South Africa [65, 66] and several viral and HIV/AIDS opportunistic diseases and infections such as bronchitis [15, 19–22], chicken pox [50], diarrhoea [17, 34, 37, 44, 113], measles [14, 20, 38], pneumonia [14], shingles [15], and venereal diseases [44]. Despite significant advances in the utilization of* L. javanica* in southern Africa over the years for numerous viral diseases (Table 1), very little antiviral evaluations have been done on crude extracts and purified compounds of the species. There is need, therefore, for more pharmacological research as* L. javanica* could be harbouring potent (RT) inhibitors which could be useful for the development of new pharmaceutical products important for use against viral diseases and infections.

### 4.9. Pesticidal Effects

Magano et al. [95] evaluated the repellent effects of hexane extracts of* L. javanica *essential oil using the in vitro tick climbing repellency bioassay on adults of* Hyalomma marginatum rufipes* Koch ticks. The authors found that 107 mg/mL caused repellency index of 100% at one hour and 30 minutes. In a similar study by Madzimure et al. [120],* L. javanica *aqueous leaf extracts at 10% and 20% w/v were effective at controlling cattle ticks (*Amblyomma* species,* Boophilus* species,* Hyalomma* species,* Rhipicephalus appendiculatus,* and* Rhipicephalus evertsi evertsi*) and were as good as the positive control amitraz-based acaricide Trickbuster®. These authors found no parasites on microscopic examination of the Giemsa-stained thin blood smear collected from treated cattle implying that the animals did not suffer from clinical tick-borne diseases. Similarly, Nyahangare et al. [59] tested the pesticidal activity of* L. javanica* water extracts against cattle ticks. The authors found no significant difference between cattle treated with a commercial synthetic acaricide and those under* L. javanica *treatment. Martinez-Velazquez et al. [166] evaluated pesticidal effects of two essential oils, namely, *γ*-terpinene** 82** and *ρ*-cymene** 99,** isolated from* Lippia graveolens* Kunth against 10-day-old* Rhipicephalus (Boophilus) microplus *(Canestrini) tick larvae using the larval packet test bioassay. The two essential oils produced high mortality ranging from 90 to 100% in all tested concentrations of 20 to 1.25% (v/v). Therefore,* L. javanica* can provide an effective tick control option where synthetic products are unavailable or unaffordable particularly in remote rural areas in sub-Saharan Africa.* Lippia javanica* is popular for tick control and management among resource-poor smallholder farmers in South Africa [58, 95] and Zimbabwe [59, 60, 120]. While these preliminary evaluations may serve as confirmation that* L. javanica* has some bioactivities against ticks, a comprehensive method of tick control is required for the resource-constrained smallholder farmers based on ethnopharmacological properties of* L. javanica*.

McGaw et al. [146] screened* L. javanica* for the anthelmintic test systems using the free living nematode* Caenorhabditis elegans*. The crude ethanol and hexane extracts showed some activity at a concentration of 2 mg/mL, with the 7-day incubation assay appearing to be more sensitive than the shorter assay. Earlier research by Mwangi et al. [1] indicated that* L. javanica *was active against* Aedes aegypti *larvae and* Sitophilus zeamais *Motschulsky (maize weevil). Katsvanga and Chigwiza [147] reported that* L. javanica* is an effective natural pesticide which can be used to control aphid species* (Brevicoryne brassicae)*. In their study, Katsvanga and Chigwiza [147] found that 1 : 1 powdered aqueous leaf extract of* L. javanica* reduced* B. brassicae* by 53.2% against 78.3% and 96.7% of two synthetic pesticides, Aphid kill and Bexadust “L,” respectively. Chikukura et al. [149] found* L. javanica* powdered leaf extracts to have insecticidal properties with potential to control grain damage by 21–33%. Mashela et al. [148] reported that the application of ground* L. javanica* leaves to soil in nematode-infested* (Meloidogyne incognita)* pots reduced the nematode numbers by 79–92% and significantly increased fresh fruit yield, dry shoot mass, plant height, and stem diameter of the tomato plants, as well as levels of potassium, nitrogen, and manganese in leaf tissue. Muzemu et al. [150] evaluated water extracts of* L. javanica* leaf powder for pesticidal effects against rape (*Brassica napus* L.) aphids,* B. brassicae,* and tomato (*Solanum lycopersicum* Lam.) red spider mites, and* Tetranychus evansi* as alternatives to conventional pesticides.* Lippia javanica *reduced* B. brassicae* and* Tetranychus evansi *by 12.5% and 63%, respectively [150]. The study demonstrated that* L. javanica* has pesticidal effects on* B. brassicae* and* T. evansi*. The reduced number of* B. brassicae* and* T. evansi* could be due to* L. javanica*'s extracts having repellent, toxic, and antifeedant effects since the species has essential oils with pesticidal properties [2]. Therefore, these findings indicate that* L. javanica *has both nematicidal and plant growth-promoting properties.

### 4.10. Toxicity and Cytotoxicity Activity

*Lippia javanica* is known to cause liver damage and photosensitisation in livestock, resulting in stock losses [167]. Triterpenoids isolated from the genus* Lippia* are icterogenic and cause jaundice as a result of liver damage [167]. The characteristic swelling, yellowing, and later peeling of unpigmented skin are due to the presence of phylloerythrin, a photodynamic porphyrin that reacts with sunlight and causes severe cell damage [167]. The compound is normally formed when chlorophyll is broken down by microorganisms in the rumen, but it now accumulates in the liver as a result of the damage caused by triterpenoids [167]. In view of the known toxicity of* Lippia* species, the prolonged use of high doses of* L. javanica* is potentially harmful [167].* Lippia javanica* showed low toxicity after 48 h exposure with the percentage of mortality below 50% [74].

Ayuko et al. [79] evaluated the toxicity of* L. javanica* using a brine shrimp cytotoxicity assay with LC_50_ value of 1138 ± 1.33 *μ*g/mL. Ayuko et al. [79] found the cytotoxicity to antiplasmodial activity ratios for the methanolic extracts of the two tested strains to be 843.0 and 650.3, and since these are greater than 100, it may be concluded that the extracts are of low toxicity. Samie et al. [73] demonstrated that a pure compound piperitenone** 162** isolated from* L. javanica *essential oil has low cytotoxicity activity against intestinal adenocarcinoma cells (i.e., the HCT-8 monolayers with IC_50_ of 265.6 ± 5.3 *μ*g/mL). Lukwa [64] evaluated the toxicity of* L. javanica* aqueous leaf extracts using sexually mature BALB/c mice with the placebo as control. Within 48 hours, all mice fed with the* L. javanica* leaf aqueous extract at 12.5–37.5% v/v were lethargic, and the overall mortality was 37.5%. Previous research by Manenzhe et al. [2] showed that hydrodistillation of* L. javanica* leaves, flowers, and stems produced oil that was poisonous against* P. falciparum *when diluted to 1% (v/v). These findings imply that, despite its apparent safety, water extracts of* L. javanica* leaves may have deleterious health implications on humans and animals if consumed at very high doses.

Many compounds that have been isolated from* L. javanica* including phenolic glycosides, flavonoids, and essential oils are not known to have acute toxic properties with the exception of icterogenin** 173. **Icterogenin** 173 **has been shown to inhibit biliary excretion in rabbits [168]. Reports in literature indicate that the consumption of xanthine** 22 **has resulted in mammalian toxicity. According to Madzimure et al. [64], xanthine** 22 **is a demethylated derivative of caffeine with pharmacological actions such as central nervous system (CNS) stimulation, relaxation of smooth muscle (especially bronchial muscle), myocardial stimulation, peripheral vasoconstriction, and diuresis. Considering the widespread use of* L. javanica* as herbal tea and medicine, it is important to determine if any toxicological effects can occur from its chronic or subchronic usage.

### 4.11. Other Activities

Mpofu et al. [169] determined the effect of inclusion of* L. javanica* leaf meal in broiler diets on growth performance, carcass characteristics, and fatty acid profiles. The authors found that the* L. javanica* fed broilers had higher total polyunsaturated fatty acids and n-3 fatty acids. The findings from the study showed that inclusion of* L. javanica* in broiler diets at 5 g/kg feed has positive influences on growth performance, carcass characteristics, and fatty acid profiles of broiler meat and, therefore,* L. javanica* has potential as growth-promoting feed additive in broilers. Samie et al. [73] evaluated antiamoebic activity of a pure compound piperitenone** 162** isolated from* L. javanica *essential oil against* Entamoeba histolytica* using microdilution method with metronidazole as the positive control, diluents (i.e., culture medium with an appropriate concentration of dimethyl sulphoxide as the negative control), and a blank (i.e., culture medium without dimethyl sulphoxide). Samie et al. [73] demonstrated that piperitenone** 162** had marked antiamoebic activity with IC_50_ value of 25 *μ*g/mL. More research is required as* L. javanica *could be harbouring potent antiamoebic properties which could be useful for managing amoebiasis, an infection that remains a significant cause of morbidity and mortality worldwide.

## 5. Conclusion

*Lippia javanica* has been used in African and Asian countries as herbal tea and medicine for many centuries. Utilization of* L. javanica* because of its flavour and medicinal properties forms the basis of the current demand for the plant species in central, eastern, and southern Africa. Research on* L. javanica* over the past decade on health promoting properties has greatly contributed to the increased consumption of the species as herbal or recreational tea. The focus of this research has been on phytochemical compounds, particularly phenolic content and antioxidant and free radical scavenging activities. Phenolic compounds present in* L. javanica* are largely responsible for the antioxidant properties possessed by the species. More research in this regard is required and future research should focus on more comprehensive chemical characterization of both crude and pure extracts and evaluate potential for commercialization and development of nutraceutical products based on traditional uses of* L. javanica. *Most of the pharmacological researches conducted on* L. javanica* so far have focused on the phytochemistry and biological properties of leaves, and little or no research has been done on roots and other plant parts. Therefore, future research on the species should focus on other plant parts, for example, flowers, roots, and stems, as well as organ-to-organ, age, and seasonal variation evaluations in the phytochemical content and pharmacological activities of the species.

The recent increase in the demand for* L. javanica *products may partly be ascribed to growing body of scientific evidence indicating important health benefits.* Lippia javanica *is widely sold as herbal tea in Botswana, South Africa, and Zimbabwe. Leaves and stems of* L. javanica* are also sold as herbal medicines in the medicinal plant “muthi” markets in South Africa [35, 170]. For local people who rely on herbal medicines as part of their primary healthcare as well as cultural beliefs, they prefer* L. javanica* harvested from the wild and unprocessed plant parts sold in informal medicinal “muthi” markets [170]. In the past, there were no records of overexploitation of* L. javanica* wild populations in southern Africa, resulting in Raimondo et al. [171] listing the species as Least Concern (LC) under the IUCN Red List Categories and Criteria version 3.1 of threatened species (http://www.iucnredlist.org). Recently, signs of overharvesting have been noted, where local people or plant traders have uprooted whole plants to supply medicinal plant “muthi” markets or use the plants as herbal medicines, brooms, or herbal tea. Therefore, large-scale commercial utilization of* L. javanica* is not sustainable if the species is harvested from the wild. Currently,* L. javanica* is cultivated on a commercial scale in Kenya [28] and South Africa [117] for essential oil production for the mosquito-repellent candles and perfume industry. Cultivation of* L. javanica *is therefore, a solution to the sustainability problems associated with harvesting of the species from the wild, and this option is also necessary for establishing commercial scale medicinal production and processing and trade enterprises. The success of commercial cultivation of* L. javanica *will depend on how the species is marketed as herbal tea and medicine and source of essential oil and health care products and as a source of functional foods.

Significant research has been made in the past 50 years into the chemistry and pharmacology of* L. javanica.* These studies have shown* L. javanica* to display various chemical and different biological activities some of which justify its ethnopharmacological utilization in variety of cultures. In the light of the evidence that* L. javanica* is combined with other plant species in traditional medicine, it will be valuable to investigate the possibility of synergistic effects of the different extracts. Deep phytochemical studies of* L. javanica* and its phytochemical properties, especially the mechanisms of action of its bioactive constituents to illustrate the correlation between ethnomedicinal uses and pharmacological activities, should be the focus of further research on the species. There is need for extensive in vivo experiments to validate the existing pharmacological activities. However, because* L. javanica* contains potentially toxic compounds, its toxicological properties need to be properly established via proper quality control of product development to ensure that potentially toxic components are kept below tolerance levels.

## Figures and Tables

**Figure 4:**
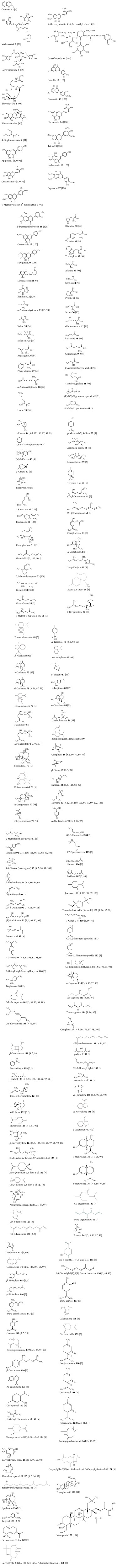


**Table 1:** Traditional uses of *Lippia javanica* in central, eastern, and southern Africa.

Use	Plant part(s) used	Mode of use	Country practised	Reference(s)
*Food uses*				
Food additive	Leaves, twigs	Leaves and twigs boiled together with maize, cassava, groundnuts, and common tea (*Camellia sinensis *(L.) Kuntze)	Kenya	[6]
Herbal tea	Leaves	Leaves can be sun dried for later use	Botswana, South Africa, and Zimbabwe	[7–11]
Leafy vegetable	Leaves	Eaten as vegetable with meat or fish	India	[12, 13]
*Respiratory problems*				
Asthma	Leaves, twigs	Decoction taken orally or smoke inhaled	South Africa, Zimbabwe	[14–17]
Blocked nose	Leaves	Leaf decoction of *L. javanica* alone taken orally or mixed with any of these species: *Brachylaena uniflora *Harv., *Clausena anisata *(Willd). Hook.f. ex Benth., *Clematis brachiata *Thunb., *Combretum molle *R. Br. ex G. Don,* Eucalyptus grandis *W. Hill, *Helichrysum kraussii *Sch. Bip., *Krauseola mosambicina *Pax & Hoffm., *Plectranthus neochilus* Schltr., *Senecio serratuloides *DC.,or *Trichilia emetica *Vahl taken orally	South Africa	[18]
Bronchitis	Leaves, roots, and stems	Leaf infusion of *L. javanica* alone taken orally or applied to skin as ointment or leaf and stem infusion of *L. javanica* mixed with leaves of *Artemisia afra* Jacq. ex Willd. taken orally	Botswana, South Africa	[15, 19–22]
Chest pains	Leaves, roots	Leaves and roots of *L. javanica* alone chewed or decoction taken orally or used in washes and poultices or smoke inhaled or “imbiza,” decoction prepared from* L. javanica* and *Cyrtanthus obliquus* (L. f.) Aiton, taken orally or leaf decoction of *L. javanica* mixed with leaves of any of these species: *Brachylaena transvaalensis *Hutch. ex E. Phillips & Schweick., *C. brachiata, E. grandis,H. kraussii, *or* T. emetica* taken orally or leaf juice taken orally with cloves of *Allium sativum *L.	Bangladesh, Ethiopia, and South Africa	[14, 18, 23–27]
Colds	Leaves, twigs	Leaf infusion of *L. javanica* alone taken orally or inhaled or applied as an ointment or “imbiza,” decoction prepared from* L. javanica* and *C. obliquus,* taken orally	Botswana, Kenya, South Africa, and Zimbabwe	[15, 16, 19, 21, 27–32]
Cough	Leaves, stems, and twigs	Leaves of *L. javanica* alone chewed or infusion inhaled or taken orally or applied as an ointment or leaf and stem infusion of *L. javanica* mixed with leaves of any of these species: *Acanthospermum glabratum *(DC.) Wild., *A. afra, B. uniflora, B. transvaalensis, Bridelia cathartica *Bertol., *C. anisata, C. brachiata, C. molle, E. grandis, H. kraussii, K. mosambicina*, *P. neochilus*, *Psidium guajava *L., *S. serratuloides, Terminalia sericea *Burch. ex DC., *Tetradenia riparia *(Hochst.) Codd, or *T. emetica *taken orally	Botswana, Ethiopia, South Africa, and Zimbabwe	[15, 16, 18–21, 26, 30, 32]
Influenza	Leaves, roots	Decoction taken orally	Mozambique, South Africa	[7, 33–36]
Lung infections	Leaves	Infusions taken orally	South Africa	[37]
Measles	Leaves, stems	Leaf decoction of *L. javanica* alone taken orally or body washed with infusion or leaf and stem infusion of *L. javanica *mixed with leaves of *A. afra *taken orally	Kenya, South Africa, and Zimbabwe	[14, 20, 38]
Pneumonia	Leaves	Ointment rubbed on chest and abdomen	Zimbabwe	[14]
Respiratory disorders	Leaves	Decoction taken orally	India, South Africa	[39–41]
Runny nose	Leaves, roots	Leaf and root decoction of *L. javanica* alone taken orally or leaf decoction of *L. javanica* mixed with leaves of any of these species: *B. transvaalensis, B. cathartica,C. anisata, C. brachiata, E. grandis, Hypoxis *spp., *K. mosambicina; P. guajava, S. serratuloides, P. neochilus, *or* T. emetica *taken orally	South Africa	[18]
Shortness of breath (dyspnoea)	Leaves	Decoction taken orally or body washed with decoction	Zimbabwe	[14]
Sore throat	Leaves	Leaf decoction of *L. javanica* alone taken orally or leaf decoction of *L. javanica* mixed with leaves of *E. grandis *or *T. riparia *taken orally	South Africa	[18]
Tonsillitis	Leaves	Leaf decoction of *L. javanica* alone taken orally or leaf decoction of *L. javanica *mixed with leaves of *E. grandis *taken orally	South Africa	[18]
Tuberculosis	Leaves	Leaf decoction of *L. javanica* alone taken orally or “imbiza,” decoction prepared from* L. javanica* and *C. obliquus,* taken orally	South Africa, Uganda	[24, 42, 43]
*Gastrointestinal diseases*				
Amoebiasis	Leaves, twigs	Decoction taken orally	Kenya	[6]
Anthelmintics	Leaf	Infusions taken orally	South Africa	[40]
Diarrhoea	Leaves, roots	Decoction taken orally	Kenya, Mozambique, South Africa	[17, 29, 34, 37, 44]
Gangrenous rectitis	Leaves	Infusions taken orally	South Africa	[45]
Prophylactic against diarrhoea	Leaves	Infusion taken orally	South Africa	[40]
Vomiting	Leaves	Decoction taken orally	Zanzibar, Tanzania	[46]
*Fever, malaria, and as insect repellent*				
Fever	Leaves, stems, and twigs	Leaf and twig decoction of *L. javanica* alone taken orally or leaf and stem infusion of *L. javanica* mixed with leaves of any of these species: *A. afra, B. transvaalensis, C. anisata*, *C. molle, E. grandis,P. guajava, S. serratuloides, T. riparia, *or *T. emetica taken *orally	South Africa, Zimbabwe	[14, 16, 18, 20]
Getting rid of lice, insects, and lice and as mosquito repellent	Leaves, twigs, and whole plant	Used in washes and poultices or as steam or sprayed or burnt to chase away mosquitoes	Ethiopia, South Africa, and Zimbabwe	[4, 26, 34, 47–51]
Malaria	Leaves, roots	Decoction taken orally	Mozambique, South Africa	[17, 33, 52]
Prophylactic against malaria	Leaves	Infusion taken orally	South Africa	[40]
*Wounds, injuries, pain, and skin infections*				
Abdominal pains	Leaves	Leaves chewed and juice swallowed	Zimbabwe	[14]
Acne	Leaves	Not specified	Botswana	[22]
Antidotes	Roots	Used as antidote for food poisoning	Botswana	[22]
Backache	Roots	Infusion taken orally	South Africa, Zimbabwe	[14, 20]
Bleeding from the nose (epistaxis)	Leaves	Fresh leaves inserted into nose or powdered leaves sniffed	South Africa, Zimbabwe	[14, 53]
Boils	Leaves	Infusion taken orally	South Africa	[54]
Chicken pox	Leaves	Used in washes and poultices or as steam	South Africa	[50]
Earache	Leaves	Decoction taken orally mixed with leaves of *E. grandis*	South Africa	[18]
Febrile rashes	Leaves	Infusions taken orally	South Africa	[40]
Headache, migraine	Leaves, roots	Leaf and root decoction of *L. javanica* alone taken orally or leaf decoction of *L. javanica *mixed with leaves of any of these species: *B. cathartica,C. brachiata, E. grandis, T. riparia, *and *T. emetica *taken orally	Kenya, South Africa, and Zimbabwe	[14, 18, 33, 34, 38]
Inflammation	Leaves	Not specified	South Africa	[41]
Pubic sores	Leaves	Decoction taken orally mixed with leaves of *Acanthospermum australe* (Loefl.) Kuntze	Swaziland	[55]
Scabies	Leaves, roots	Infusion taken orally or used in washes and poultices or as steam	South Africa, Zimbabwe	[14, 50, 54]
Shingles	Leaves, twigs, and roots	Applied as an ointment	South Africa	[15]
Skin disorders, such as heat rash scratches, stings, and bites	Leaves, twigs	Applied as an ointment or “imbiza,” decoction prepared from* L. javanica* and *C. obliquus,* taken orally	South Africa	[21, 27]
Sores	Leaves	Decoction of *L. javanica* mixed with bark of *Acacia burkei *Benth., *Ozoroa engleri *R. Fern. & A. Fern., *Sclerocarya birrea *(A. Rich.) Hochst., *Syzygium cordatum* Hochst. ex Krauss, and *Tabernaemontana elegans *Stapf taken orally	South Africa	[56]
Sore eyes, cataracts	Leaves, roots	Juice squeezed into eyes	Botswana, Zimbabwe	[14, 22]
Sprained joints	Roots	Root powder applied to scarifications around sprained joints	South Africa	[20]
Ulcers	Leaves	Juice taken orally with cloves of *A. sativum*	Bangladesh	[23]
Wounds	Leaves	Fresh leaves wrapped around wound to enhance healing or infusion taken orally	Kenya, South Africa	[54, 57]
*Ethnoveterinary uses*				
Disinfecting suspected anthrax-infested meat	Whole plant	Whole plants used to disinfect suspected anthrax-infested meat	South Africa	[20]
Getting rid of ticks and other ectoparasites	Leaves, twigs, and whole plant	Crushed leaves mixed with water and sprayed, twigs used as bedding in fowl runs	South Africa, Zimbabwe	[58–60]
Lice repellant	Whole plant	Whole plant used to repel lice in poultry	India	[61]
Milk preservative	Stem	Stem applied to milk gourd before milk fermentation	Kenya	[62]
*Other uses*				
Anaemia in pregnancy	Leaves	Decoction taken orally	Zanzibar, Tanzania	[46]
Broom	Whole plant	Whole plants cut and tied together to make rough brooms	South Africa	[53]
Cancer	Leaves	“Imbiza,” decoction prepared from* L. javanica* and *C. obliquus,* taken orally	South Africa	[27]
Ceremonial	Leaves, whole plant	Used before and after funerals or placed on patients' bed after circumcision	Kenya, South Africa	[31, 58, 63]
Convulsions	Leaves	Leaves rubbed on face	Zimbabwe	[14, 64]
Diabetes	Leaves	“Imbiza,” decoction prepared from* L. javanica* and *C. obliquus,* taken orally	South Africa	[27]
Fatigue or tiredness	Leaves	Leaf decoction of *L. javanica* mixed with leaves of *A. glabratum* or *E. grandis *or *T. riparia *taken orally	South Africa	[18]
Fence	Whole plant	Planted around homesteads	Ethiopia	[26]
Fodder	Leaves	Leaves eaten by goats	Kenya	[31]
Fuelwood	Whole plant	Whole plant used as fuelwood	Zanzibar, Tanzania	[46]
Human immunodeficiency virus (HIV) or acquired immunodeficiency syndrome (AIDS) symptoms	Leaves	“Imbiza,” decoction prepared from *L. javanica* and *C. obliquus* which is taken orally	South Africa	[65, 66]
Kidney problems	Root	50 g root powder boiled in 2 litres of water; patient takes a cup of this mixture once per day for 3 days	Swaziland	[67]
Madness	Leaves, whole plant	Body washed with leaf infusion	Malawi, Zimbabwe	[14, 68]
Marasmic infants	Roots	Infusions used to bath infants	South Africa, Zimbabwe	[14, 20]
Night blindness	Leaves	Face washed with infusion	Zimbabwe	[14]
Nightmares	Not specified	Not specified	Zimbabwe	[64]
Pre-, intra-, and postpartum complications	Stem, roots	Smear powder of root or stem	Kenya	[69]
Preventing odours or being used indoors to freshen air	Leaves, whole plant	Leaves sprinkled in toilets to prevent odours or whole plant placed in vase or room	Kenya, South Africa	[31, 35, 58]
Protection against dogs and crocodiles	Leaves	Leaves smeared on body	South Africa	[70]
Psychotropic behaviour	Leaves	Infusion taken orally	Swaziland	[71]
Removing bad luck	Leaves	Face and hands washed with infusion of *L. javanica* leaves mixed with *C. molle* leaves	Swaziland	[72]
Sleepless nights	Leaves	Leaf decoction of *L. javanica* mixed with leaves of *E. grandis* and *T. riparia *taken orally	South Africa	[18]
To drive away bad spirits	Leaves	Body washed with infusion	Zimbabwe	[14]
To prevent infants from contracting illness caused by father or mother committing adultery	Leaves	Leaves rubbed on forehead, elbows, and knees after committing adultery	Zimbabwe	[14]
Venereal diseases	Roots	Decoction taken orally	Mozambique	[44]
Weak joints	Leaves	Decoction taken orally	Zimbabwe	[14]

**Table 2:** Nutritional composition of *Lippia javanica* leaves.

Caloric and nutritional composition	Values	Reference
Ash	1.60 ± 0.001 g	[13]
Calorific value	50.64 ± 5.63 kcal	[13]
Crude fat	0.16 ± 0.001 g	[13]
Crude fibre	2.63 ± 0.002 g	[13]
Crude protein	1.22 ± 0.0004 g	[13]
Dry matter	15.46 ± 1.40 g	[13]
Moisture	90.44 ± 0.26 g	[13]
Total carbohydrate	6.55 ± 0.26 g	[13]
Vitamin C	22.42 ± 0.001 mg	[13]
Ca	2856–9225 *μ*g g^−1^	[27]
Mg	1598–5619 *μ*g g^−1^	[27]
Fe	75–750 *μ*g g^−1^	[27]
Mn	40.1 ± 1.16 *μ*g g^−1^	[27]
Zn	15.6–27.3 *μ*g g^−1^	[27]
Cu	3.0–8.5 *μ*g g^−1^	[27]
Cr	0–2.7 *μ*g g^−1^	[27]
Se	2.57 ± 0.19 *μ*g g^−1^	[27]
Pb	0.38–1.19 *μ*g g^−1^	[27]
Cd	0.53 ± 0.05 *μ*g g^−1^	[27]
Co	0.19 ± 0.01 *μ*g g^−1^	[27]

**Table 3:** Total phenolic content (TPC) as gallic acid equivalents (GAE) and tannin content as leucocyanidin equivalents (LE) and free radical scavenging activity (FRSA) of herbal tea extracts (after Bhebhe et al. [10]).

Plant species	TPC g GAE/100 g	Tannin content of LE/100 g	Percentage FRSA	IC_50_ g/mL
*Lippia javanica*	12.46 ± 4.31	0.12 ± 0.01	83.77 ± 0.8	0.016
*Aspalathus linearis *	6.69 ± 0.83	0.94 ± 0.04	67.27 ± 0.25	0.053
*Adansonia digitata*	0.79 ± 0.28	1.69 ± 0.01	43.07 ± 1.0	0.132
*Fadogia ancylantha*	2.88 ± 0.48	0.20 ± 0.12	70.0 ± 0.40	0.051
*Ficus sycamores*	14.02 ± 0.01	1.98 ± 0.12	86.13 ± 0.85	0.009
*Myrothamnus flabellifolius*	4.75 ± 0.33	1.04 ± 0.01	80.93 ± 0.75	0.019

**Table 4:** Summary of pharmacological activities of the extracts isolated from different parts of *Lippia javanica.*

Activity tested	Extract	Plant part	Model	Effect	Reference
Antiamoebic	Piperitenone **162**	Leaves	Microdilution assay	Active with IC_50_ value of 25 *μ*g/mL against *E. histolytica*	[73]

Antibacterial	Piperitenone **162**	Leaves	Microdilution assay	Active with MIC value of 50 *μ*g/mL against *A. calcoaceticus, M. kristinae* (50 *μ*g/mL), *S. typhi* (25 *μ*g/mL), and *S. aureus *(12 *μ*g/mL)	[73]

Antibacterial (Antiproteus)	Methanol	Leaves	Disc diffusion assay	Active withMIC value of 313 *μ*g/mL* against P. mirabilis* and 926 *μ*g/mL against *P. vulgaris *	[74]
	Water	Leaves	Disc diffusion assay	Exhibited weak activity against *P. *mirabilis with MIC value of 1873 *μ*g/mL and 1768 *μ*g/mL against *P. vulgaris *	[74]

Antibacterial	Acetone	Leaves	Microdilution assay	Active against *E. coli *withMIC value of 0.64 mg/mL, *E. faecalis *(0.64), *P. aeruginosa *(0.32), and *S. aureus *(0.64). Total activity MIC values were as follows: *E. coli *(127 mg/mL), *E. faecalis *(127 mg/mL), *P. aeruginosa *(253 mg/mL), and *S. aureus *(127 mg/mL)	[75]
	Acetone	Leaves	Microdilution assay	Active against* B. cereus with *MIC value of 3 mg/mL, *Bacillus pumilus (*6 mg/mL), *B. subtilis *(>12 mg/mL), *S. aureus* (12 mg/mL), *E. faecalis* (6 mg/mL), *E. cloacae* (6 mg/mL), *E. coli* (6 mg/mL), *Pantoea agglomerans* (12 mg/mL), *P. aeruginosa* (12 mg/mL), *Shigella flexneri* (1.5 mg/mL), *Aeromonas hydrophila* (12 mg/mL), *P. mirabilis* (>12 mg/mL), *K. pneumoniae* (12 mg/mL), *Salmonella choleraesuis* (6 mg/mL), and *Serratia marcescens *(>12 mg/mL)	[17]
	Hexane	Leaves	Microdilution assay	Active against *B. cereus with *MIC value of 12 mg/mL, *B. pumilus *(12 mg/mL), *B. subtilis* (12 mg/mL), *S. aureus* (6 mg/mL), *E. faecalis* (12 mg/mL), *E. cloacae* (12 mg/mL), *E. coli* (6 mg/mL), *P. agglomerans* (12 mg/mL), *P. aeruginosa* (12 mg/mL), *S. flexneri* (6 mg/mL), *A. hydrophila* (12 mg/mL), *P. mirabilis* (12 mg/mL), *K. pneumoniae* (12 mg/mL), *S. choleraesuis* (6 mg/mL), and *antiamoebic effect* (12 mg/mL)	[17]
	Methanol	Leaves	Microdilution assay	Active against* B. cereus with *MIC values of 1.5 mg/mL, *B. pumilus *(6 mg/mL), *B. subtilis* (>12 mg/mL), *S. aureus* (12 mg/mL), *E. faecalis* (3 mg/mL), *E. cloacae* (6 mg/mL), *E. coli* (6 mg/mL), *P. agglomerans* (12 mg/mL), *P. aeruginosa* (12 mg/mL), *S. flexneri* (1.5 mg/mL), *A. hydrophila* (6 mg/mL), *P. mirabilis* (>12 mg/mL), *K. pneumoniae* (12 mg/mL), *S. choleraesuis* (6 mg/mL), and *S. marcescens* (12 mg/mL)	[17]
	Essential oil	Leaves	Microdilution assay	Active against *B. cereus with *MIC values of 6 mg/mL, *B. pumilus* (6 mg/mL), *B. subtilis* (3 mg/mL), *S. aureus* (1.5 mg/mL), *E. faecalis* (3 mg/mL), *E. cloacae* (12 mg/mL), *E. coli* (6 mg/mL), *P. agglomerans* (6 mg/mL), *P. aeruginosa* (12 mg/mL), *S. flexneri* (3 mg/mL), *A. hydrophila* (3 mg/mL), *P. mirabilis* (>12 mg/mL), *K. pneumoniae* (3 mg/mL), *S. choleraesuis* (1.5 mg/mL), and *S. marcescens* (>12 mg/mL)	[17]
	Methanol	Leaves	Microdilution assay	Active against *E. faecalis* with MIC value of 0.14 mg/mL	[7]
	Methanol	Leaves	Microdilution assay	Active against *E. coli *with MIC value of 0.31 mg/mL	[7]
	Methanol	Leaves	Microdilution assay	Active against *P. aeruginosa* with MIC value of 0.42 mg/mL	[7]
	Methanol	Leaves	Microdilution assay	Active against *S. aureus* with MIC value of 0.13 mg/mL	[7]

Antifungal	Acetone	Leaves	Microdilution assay	Active against *C. albicans with MIC value of >7*.5 mg/mL, *C. krusei* (1.88 mg/mL), and *C. neoformans* (>7.5 mg/mL)	[7]
	Hexane	Leaves	Microdilution assay	Active against* C. albicans *with MIC value of >3.75 mg/mL, *C. krusei *(3.75 mg/mL), and *C. neoformans* (>7.5 mg/mL)	[7]
	Acetone	Leaves	Microdilution assay	Active against* C. krusei *with MFC value of 7.5 mg/mL	[7]
	Dichloromethane	Aerial parts	Microdilution assay	Active against *Fusarium proliferatum *with MIC value of 0.14 mg/mL and *Fusarium verticillioides* (0.19 mg/mL)	[76]
	Hexane	Aerial parts	Microdilution assay	Active against *F. proliferatum *with MIC value of 0.23 mg/mL and *F. verticillioides* (0.45 mg/mL)	[76]
	Methanol	Aerial parts	Microdilution assay	Active against *F. proliferatum* with MIC value of 1.77 mg/mL and *F. verticillioides* (0.43 mg/mL)	[76]
	Methanol	Aerial parts	Microdilution assay	Active against *F. proliferatum* with MIC value of >2.50 mg/mL and *F. verticillioides* (>2.50 mg/mL)	[76]

Antimycobacterial	Acetone	Leaves	Microdilution assay	Active with MIC value of 0.47 mg/mL and total activity of 10 mL/g against *M. smegmatis*	[77]
	Dichloromethane	Leaves	Microdilution assay	Active with MIC value of 1.25 mg/mL and total activity of 23 mL/g against *M. smegmatis*	[77]
	Hexane	Leaves	Microdilution assay	Active with MIC value of 0.62 mg/mL and total activity of 13 mL/g against *M. smegmatis*	[77]
	Methanol	Leaves	Microdilution assay	Active with MIC value of 1.25 mg/mL and total activity of 7 mL/g against *M. smegmatis*	[77]

Antioxidant	Water	Leaves	DPPH assay	Exhibited activity with EC_50_ value of 358 *μ*g/mL	[7]
	Water	Leaves	—	Exhibited activity with 209 ascorbic acid equivalent (mg/g dry weight)	[7]
	Water	Leaves	ABTS assay	Active with TEAC value of 1.5 mmol/100 g	[78]
	Water	Leaves	DPPH assay	Active with TEAC value of 1462.54 mmol/100 g	[78]
	Water	Leaves	FRAP assay	Active with TEAC value of 2.38 mmol/100 g	[78]

Antiplasmodial	Hexane, chloroform	Roots	Microdilution assay	Active against *P. falciparum *chloroquine sensitive with IC_50_ value of 12.25 ± 0.72 *μ*g/mL	[79]
	Ethyl acetate	Roots	Microdilution assay	Active against* P. falciparum *chloroquine sensitive with IC_50_ value of 12.12 ± 0.79 *μ*g/mL	[79]
	Methanol	Roots	Microdilution assay	Active against* P. falciparum *chloroquine sensitive with IC_50_ value of 1.35 ± 0.06 *μ*g/mL	[79]
	Hexane, chloroform	Roots	Microdilution assay	Active against* P. falciparum *chloroquine resistant with IC_50_ value of 18.59 ± 0.26 *μ*g/mL	[79]
	Ethyl acetate	Roots	Microdilution assay	Active against* P. falciparum *chloroquine resistant with IC_50_ value of 15.80 ± 0.26 *μ*g/mL	[79]
	Methanol	Roots	Microdilution assay	Active against *P. falciparum *chloroquine resistant with IC_50_ value of 1.75 ± 0.17 *μ*g/mL	[79]
	Dichloromethane	Roots	pLDH assay	Active against *P. falciparum *withIC_50_ value of 3.8 *μ*g/mL	[80]
	Dichloromethane/methanol	Roots	pLDH assay	Active against *P. falciparum *withIC_50_ value of 27 *μ*g/mL	[80]
	Methanol	Roots	pLDH assay	Active against *P. falciparum *withIC_50_ value of 24 *μ*g/mL	[80]
	Water	Roots	pLDH assay	Active against* P. falciparum with *IC_50_ value of >100 *μ*g/mL	[80]
	Dichloromethane	Stems	pLDH assay	Active against* P. falciparum *withIC_50_ value of 4.5 *μ*g/mL	[80]
	Dichloromethane/methanol	Stems	pLDH assay	Active against *P. falciparum *withIC_50_ value of 21.8 *μ*g/mL	[80]
	Methanol	Stems	pLDH assay	Active against *P. falciparum *with IC_50_ value of 29.8 *μ*g/mL	[80]
	Water	Stems	pLDH assay	Active against *P. falciparum *withIC_50_ value of >100 *μ*g/mL	[80]

Free radical scavenging activity	Water	—	DPPH assay	Active with IC_50_ value of 0.059 ± 0.02 g/mL	[11]
	50% methanol	—	DPPH assay	Active with IC_50_ value of 0.04 ± 0.001 g/mL	[11]
	Ethanol	—	DPPH assay	Active with IC_50_ value of 0.025 ± 0.001 g/mL	[11]
	50% ethanol	—	DPPH assay	Active with IC_50_ value of 0.027 ± 0.005 g/mL	[11]
	Acetone	—	DPPH assay	Active with IC_50_ value of 0.057 ± 0.004 g/mL	[11]
	50% acetone	—	DPPH assay	Active with IC_50_ value of 0.022 ± 0.001 g/mL	[11]
	Ethyl acetate	—	DPPH assay	Active with IC_50_ value of 0.066 ± 0.001 g/mL	[11]

Toxicity	Methanol	Leaf	Brine shrimp lethality assay	40% mortality recorded after 48 h exposure towards *Artemia nauplii*	[74]
	Methanol	Roots	Brine shrimp lethality assay	Active against *P. falciparum *chloroquine sensitive with IC_50_ value of 843.0 *μ*g/mL	[79]
	Methanol	Roots	Brine shrimp lethality assay	Active against *P. falciparum *chloroquine resistant with IC_50_ value of 650.3 *μ*g/mL	[79]

**Table 5:** 

Chemical compound	Reference(s)
*Phenolic compounds*	
Coumarin **1**	[4]
Verbascoside **2**	[81]
Isoverbascoside **3**	[81]
Theveside-Na **4**	[82]
Theveridoside **5**	[82]
4-ethylnonacosane **6**	[83]
Apigenin **7**	[64, 83]
Cirsimaritin **8**	[64, 83]
6-Methoxy luteolin 4′-methyl ether **9**	[83]
6-Methoxy luteolin 3′,4′,7-trimethyl ether **10**	[83]
Crassifolioside **11**	[64]
Luteolin **12**	[64]
Diosmetin **13**	[64]
Chrysoeriol **14**	[64]
Tricin **15**	[64]
Isothymusin **16**	[64]
Eupatorin **17**	[64]
5-Dimethyl noboletin **18**	[64]
Genkwanin **19**	[64]
Salvigenin **20**	[64]
Lippialactone **21**	[84]
*Alkaloid*	
Xanthine **22**	[64]
*Amino acids*	
*α*-Aminobutyric acid **23**	[85, 86]
Valine **24**	[86]
Isoleucine **25**	[86]
Asparagine **26**	[86]
Phenylalanine **27**	[86]
*α*-Aminoadipic acid **28**	[86]
Lysine **29**	[86]
Histidine **30**	[86]
Tyrosine **31**	[86]
Tryptophan **32**	[86]
Alanine **33**	[85]
Glycine **34**	[85]
Proline **35**	[85]
Serine **36**	[85]
Glutamine acid **37**	[85]
*β*-Alanine **38**	[85]
Glutamine **39**	[85]
*β*-Aminoisobutyric acid **40**	[85]
4-hydroxyproline **41**	[85]
*Essential oil*	
(E)-2(3)-tagetenone epoxide **42**	[83]
4-Methyl-2-pentanone **43**	[3]
*α*-Pinene **44**	[3–5, 87–91]
1,3-5-Cycloheptatriene **45**	[4]
(+)-2-Carene **46**	[4]
3-Carene **47**	[4]
Eucalyptol **48**	[4]
1.8 myrcene **49**	[87]
Ipsdienone **50**	[87]
Caryophyllene **51**	[85]
Geranial **52**	[5, 92, 93]
2,6-Dimethylstyrene **53**	[92]
Geraniol **54**	[92]
Octen-3-one **55**	[5]
6-Methyl-5-hepten-2-one **56**	[5]
*ρ*-Mentha-1(7),8-diene **57**	[5]
Artemisia ketone **58**	[5]
Linalool oxide **59**	[5]
Terpinen-4-ol **60**	[5]
(Z)-*β*-Ocimenone **61**	[5]
(E)-*β*-Ocimenone **62**	[5]
Carvyl acetate **63**	[5]
*α*-Cubebene **64**	[5]
Sesquithujene **65**	[5]
Acora-3,5-diene **66**	[5]
*β*-Bergamotene **67**	[5]
Trans-calamenene **68**	[5]
*β*-Alaskene **69**	[5]
*γ*-Cadinene **70**	[5]
*δ*-Cadinene **71**	[3, 88, 89, 91]
Cis-calamenene **72**	[5]
Nerolidol **73**	[5]
*(E)*-Nerolidol **74**	[3, 88, 89]
Spathulenol **75**	[5]
Epi-*α*-muurolol **76**	[5]
*α*-Longipinene **77**	[90]
Chrysanthenone **78**	[90]
*α*-Terpineol **79**	[3, 5, 90, 91]
*α*-Amorphene **80**	[90]
*α*-Thujene **81**	[91]
*γ*-Terpinene **82**	[91]
*α*-Cubebene **83**	[91]
Linalool acetate **84**	[91]
Bicyclosesquiphellandrene **85**	[91]
Camphene **86**	[3, 5, 88–91]
*β*-Pinene **87**	[3, 5, 91]
Sabinene **88**	[3, 5, 87, 90, 91]
Myrcene **89**	[3, 5, 87–89, 91–95]
*α*-Phellandrene **90**	[3, 5, 88, 89]
2-Methylbutyl isobutyrate **91**	[3]
Limonene **92**	[3, 5, 88–94]
1,8-Cineole **93**	[3, 5, 90, 91, 95]
*β*-Phellandrene **94**	[3, 4, 88, 89, 91]
*(Z)*-3-Hexenal **95**	[3]
*(Z)*-*β*-Ocimene **96**	[3, 5, 88, 89, 91]
*(E)*-*β*-Ocimene **97**	[3, 5, 88, 89, 91]
Isomyrcenol **98**	[3]
*p*-Cymene **99**	[3, 5, 85, 88–91]
2-Methylbutyl-2-methyl butyrate **100**	[3]
Terpinolene **101**	[3]
Dihydrotagetone **102**	[3, 88, 89, 91, 95]
*Cis*-alloocimene **103**	[3, 88, 89]
*(Z)*-3-Hexen-1-ol **104**	[3]
6,7-Epoxymyrcene **105**	[3]
Nonanal **106**	[3]
Perillene **107**	[3, 91]
Ipsenone **108**	[3, 87–89, 95]
*Trans*-Linalool oxide *(furanoid) * **109**	[3, 88–90]
1-Octen-3-ol **110**	[3, 88, 89]
*Cis*-1,2-limonene epoxide **111**	[3]
*Ttrans*-1,2-limonene epoxide **112**	[3]
*cis*-Linalool oxide *(furanoid) * **113**	[3, 88–90]
*α*-Copaene **114**	[3, 5, 88–90]
*Cis*-tagetone **115**	[3, 88, 89]
*Trans*-tagetone **116**	[3, 88, 89]
Camphor **117**	[3, 5, 88–90, 93, 94]
*β*-Bourbonene **118**	[3, 5, 91]
Benzaldehyde **119**	[3, 5]
Linalool **120**	[3, 5, 85, 88–90, 92, 93]
*Trans*-*α*-bergamotene **121**	[3]
*α*-Cedrene **122**	[3, 5]
Myrcenone **123**	[3, 5, 83, 91]
*β*-Caryophyllene **124**	[3, 5, 87–91, 93, 94]
2-Methyl-6-methylene-3,7-octadien-2-ol **125**	[3]
*Trans*-*p*-mentha-2,8-dien-1-ol **126**	[3]
*Cis*-*p*-mentha-2,8-dien-1-ol **127**	[3]
Alloaromadendrene **128**	[3, 5, 88, 89]
*(Z)*-*β*-Farnesene **129**	[3]
*(E)*-*β*-Farnesene **130**	[3, 5]
*(E*,*E)*-*α*-Farnesene **131**	[3, 88, 89]
Ipsdienol **132**	[3]
*(Z)*-3-Hexenyl tiglate **133**	[3]
Isovaleric acid **134**	[3]
*α*-Humulene **135**	[3, 5, 88, 89, 91]
*α*-Acoradiene **136**	[3]
*β*-Acoradiene **137**	[3]
*γ*-Muurolene **138**	[3, 5, 88, 89]
*α*-Muurolene **139**	[3, 5, 88–90]
*Cis*-tagetenone **140**	[3]
*Trans*-tagetenone **141**	[3]
Borneol **142**	[3, 5, 88–90]
Verbenone **143**	[3, 91]
Germacrene-D **144**	[3, 87–89, 93]
*β*-Bisabolene **145**	[3, 5]
*γ*-Bisabolene **146**	[3]
*Trans*-carvyl acetate **147**	[3]
Carvone **148**	[3, 5, 91]
Bicyclogermacrene **149**	[3, 5, 88, 89, 91]
*β*-Curcumene **150**	[3]
Ar-curcumene **151**	[3]
*Cis*-piperitol **152**	[3]
2-Methyl-2-butenoic acid **153**	[3]
*Trans*-*p*-mentha-1(7),8-dien-2-ol **154**	[3]
*Cis*-*p*-mentha-1(7),8-dien-2-ol **155**	[3]
2,6-Dimethyl-3*(E)*,5*(E)*,7-octatriene-2-ol **156**	[3, 88, 89]
*Trans*-carveol **157**	[3]
Calamenene **158**	[3]
Carvone oxide **159**	[3]
Isopiperitenone **160**	[3]
*Cis*-carveol **161**	[3]
Piperitenone **162**	[2, 3, 73, 83]
Isocaryophyllene oxide **163**	[3, 88, 89]
Caryophyllene oxide **164**	[3–5, 88–90]
Humulene epoxide II **165**	[3, 5, 88, 89]
Hexahydrofarnesyl acetone **166**	[3]
Spathulenol **167**	[3]
Eugenol **168**	[3, 5]
Germacrene-D-4-ol **169**	[3]
Caryophylla-2(12),6(13)-dien-5*β*-ol (=*Caryophylladienol I) * **170**	[3]
Caryophylla-2(12),6(13)-dien-5*α*-ol (=*Caryophylladienol II) * **171**	[3]
Euscaphic acid **172**	[83]
Icterogenin **173**	[96]

## Appendix

### A. Chemical Compounds Isolated and Characterized from* Lippia javanica*

See Table 5.

### B. Chemical Structures of Phenolic Compounds, Alkaloids, Amino Acids, and Essential Oils Isolated from* Lippia javanica*

See Figure 4.
